# Targeting mitochondrial bioenergetics as a promising therapeutic strategy in metabolic and neurodegenerative diseases

**DOI:** 10.1016/j.bj.2022.05.002

**Published:** 2022-05-11

**Authors:** Gurjit Kaur Bhatti, Anshika Gupta, Paras Pahwa, Naina Khullar, Satwinder Singh, Umashanker Navik, Shashank Kumar, Sarabjit Singh Mastana, Arubala P. Reddy, P. Hemachandra Reddy, Jasvinder Singh Bhatti

**Affiliations:** a Department of Medical Lab Technology, University Institute of Applied Health Sciences, Chandigarh University, Mohali Punjab, India; b Department of Biotechnology, Sri Guru Gobind Singh College, Chandigarh, India; c Department of Zoology, Mata Gujri College, Fatehgarh Sahib, Punjab, India; d Department of Computer Science and Technology, Central University of Punjab, Bathinda, India; e Department of Pharmacology, Central University of Punjab, Bathinda, India; f Department of Biochemistry, School of Basic Sciences, Central University of Punjab, Bathinda, India; g School of Sport, Exercise and Health Sciences, Loughborough University, Loughborough, UK; h Department of Nutritional Sciences, Texas Tech University, Lubbock, TX, USA; i Department of Internal Medicine, Texas Tech University Health Sciences Center, Lubbock, TX, USA; j Department of Pharmacology and Neuroscience, Texas Tech University Health Sciences Center, Lubbock, TX, USA; k Department of Public Health, Graduate School of Biomedical Sciences, Texas Tech University Health Sciences Center, Lubbock, TX, USA; l Department of Neurology, Texas Tech University Health Sciences Center, Lubbock, TX, USA; m Department of Speech, Language, and Hearing Sciences, Texas Tech University Health Sciences Center, Lubbock, TX, USA; n Department of Human Genetics and Molecular Medicine, School of Health Sciences, Central University of Punjab, Bathinda, India

**Keywords:** Bioenergetics, Mitochondrial dysfunction, Oxidative stress, Electron transport chain, Antioxidants

## Abstract

Mitochondria are the organelles that generate energy for the cells and act as biosynthetic and bioenergetic factories, vital for normal cell functioning and human health. Mitochondrial bioenergetics is considered an important measure to assess the pathogenesis of various diseases. Dysfunctional mitochondria affect or cause several conditions involving the most energy-intensive organs, including the brain, muscles, heart, and liver. This dysfunction may be attributed to an alteration in mitochondrial enzymes, increased oxidative stress, impairment of electron transport chain and oxidative phosphorylation, or mutations in mitochondrial DNA that leads to the pathophysiology of various pathological conditions, including neurological and metabolic disorders. The drugs or compounds targeting mitochondria are considered more effective and safer for treating these diseases. In this review, we make an effort to concise the available literature on mitochondrial bioenergetics in various conditions and the therapeutic potential of various drugs/compounds targeting mitochondrial bioenergetics in metabolic and neurodegenerative diseases.

## Introduction

Mitochondria are the double membrane-bound organelles of a cell, producing energy in Adenosine Triphosphate (ATP). During the process of ATP generation, several kinds of reactive oxygen species (ROS) are generated. So, the mitochondria are the primary source of ROS in the cells. Under normal conditions, ROS are continually developed and neutralized in the cells, and a balance of prooxidants and antioxidants is created. A mitochondrion, which hosts most of the oxidation pathways, is loaded with different redox centres and carriers, which can leak single electrons to oxygen and turn it into the ROS progenitor, a superoxide anion [1], and this can be further converted into harmful oxidants causing oxidative stress that can affect the mitochondrial function of cells and become one of the critical factors behind several diseases. Mitochondrial dynamics is a strictly controlled mechanism that regulates a cell's mitochondrial density and can be changed according to its physiological state. The alterations in the fission or fusion (mitochondrial dynamics), modifications in the size and structure, mutations of mtDNA or nuclear mitochondrial genes, bioenergetics defects, and other factors can cause mitochondrial dysfunction, which may further support the development of several diseases [2]. Mitochondria dysfunction included abnormalities in mitochondrial morphology, excess generation of ROS, oxidative stress, impairment of oxidative phosphorylation, and mutations in mitochondrial DNA. It may lead to the initiation of apoptosis in the cells. Since ATP is generated in mitochondria via an electron transport chain (ETC), the correct functioning of the chain is essential for healthy cells. The ETC is divided into two processes: electron transmission and proton gradient formation across the membrane. Dysfunction of various complexes in the ETC may lead to several diseases either through genetic or exogenous factors. Since the mitochondria have gained importance in a cell's normal functioning, they might be used as promising therapeutic strategies in conditions like metabolic, neurodegenerative, and cancer. Several drugs and compounds targeting mitochondria have been studied or are still under investigation. This article reviews the primary bioenergetics defects in different diseases and targets mitochondrial bioenergetics to treat the associated neurological and metabolic disorders.

## Mitochondrion: structure and functions

Mitochondria are highly dynamic eukaryotic organelles and producers of the energy currency (ATP) of an organism. These double-membrane organelles are crucial to a cell, providing many functions other than ATP production, such as cell cycle regulation, apoptosis, calcium signalling, free-radical scavenging, regulating the basic metabolism [3]. The inner lipid membrane comprises of different morphological regions: the membrane boundary, which is tightly connected to the outer membrane, the cristae, and the cristae junctions. Electron gradient is formed as the outer membrane is more permeable to ions than the inner membrane. The outer membrane also consists of enzymes essential for ATP production [2]. However, the inner membrane consists of infoldings called cristae elongated to the matrix for increasing surface area; ETC proteins are also located here. The outer membrane is more porous and permeable to low molecular weight substances and ions. Each mitochondrion consists of a double-stranded circular DNA called mitochondrial DNA (mtDNA) that encodes for some of the subunits of ETC complexes I, III, IV, and V. The process of ATP production or oxidative phosphorylation through ETC is explained in next section.

**Fig. 1:**
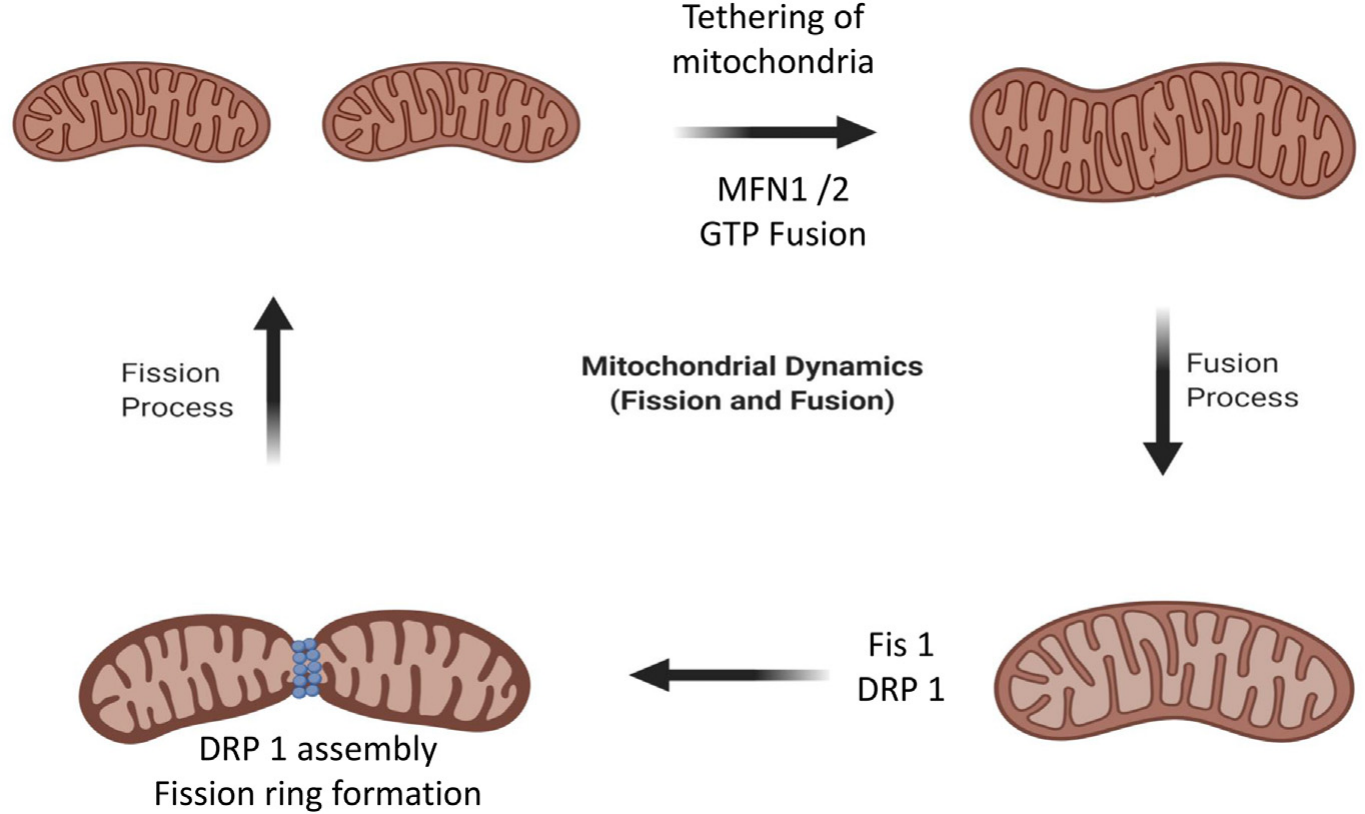
**Process of mitochondrial fission and fusion in cells.** The mitochondrial fusion and fission are regulated by guanosine triphosphatases (GTPases) of dynamin family. The fusion is conducted by mitofusin 1 and 2 (MFN1 and MFN2) and optic atrophy 1 (OPA1) proteins, while Fission 1 (FIS1) and Dynamin-related protein 1 (DRP1) help in the regulation of fission. The mitofusins (Mfn1 and Mfn2) mediate the fusion of outer membranes, and OPA1 mediates the fusion of inner membranes of a mitochondrion, which results in an elongated mitochondrion DRP1 is transported from cytosol to the outer mitochondrial membrane, making an assembly on the surface which results in separation of the organelle into two.

Being highly active organelles, mitochondria undergo organized cycles of two processes: fusion and fission, to manage their structure, distribution, and size. This is collectively referred to as mitochondrial dynamics, including internal trafficking and mitophagy, and is necessary for maintaining correct mitochondrial number and morphology [4]. In the process of mitochondrial fission, which is mediated by GTPase protein dynamin-related protein 1 (DRP1), a mitochondrion, just like a cell that grows before division, elongates and grows in volume before septation, and this result in the formation of two separate daughter mitochondria. Contrary, the process of mitochondrial fusion involves the merging of two mitochondria together with the help of GTPase proteins, mitofusin 1 and 2 (MFN1 and MFN2) lying in the outer membrane and optic atrophy 1 (OPA1) in the inner membrane through tethering. Mitochondrial fission involves the division of mitochondria and the separation of the part of a mitochondrion that has accumulated damage and debris over a long time, followed by mitophagy, i.e., degradation of mitochondria by autophagy [5]. Fig. 1 shows the mitochondrial dynamics involving fusion and fission of mitochondria.

## Mitochondrial bioenergetics

**Fig. 2:**
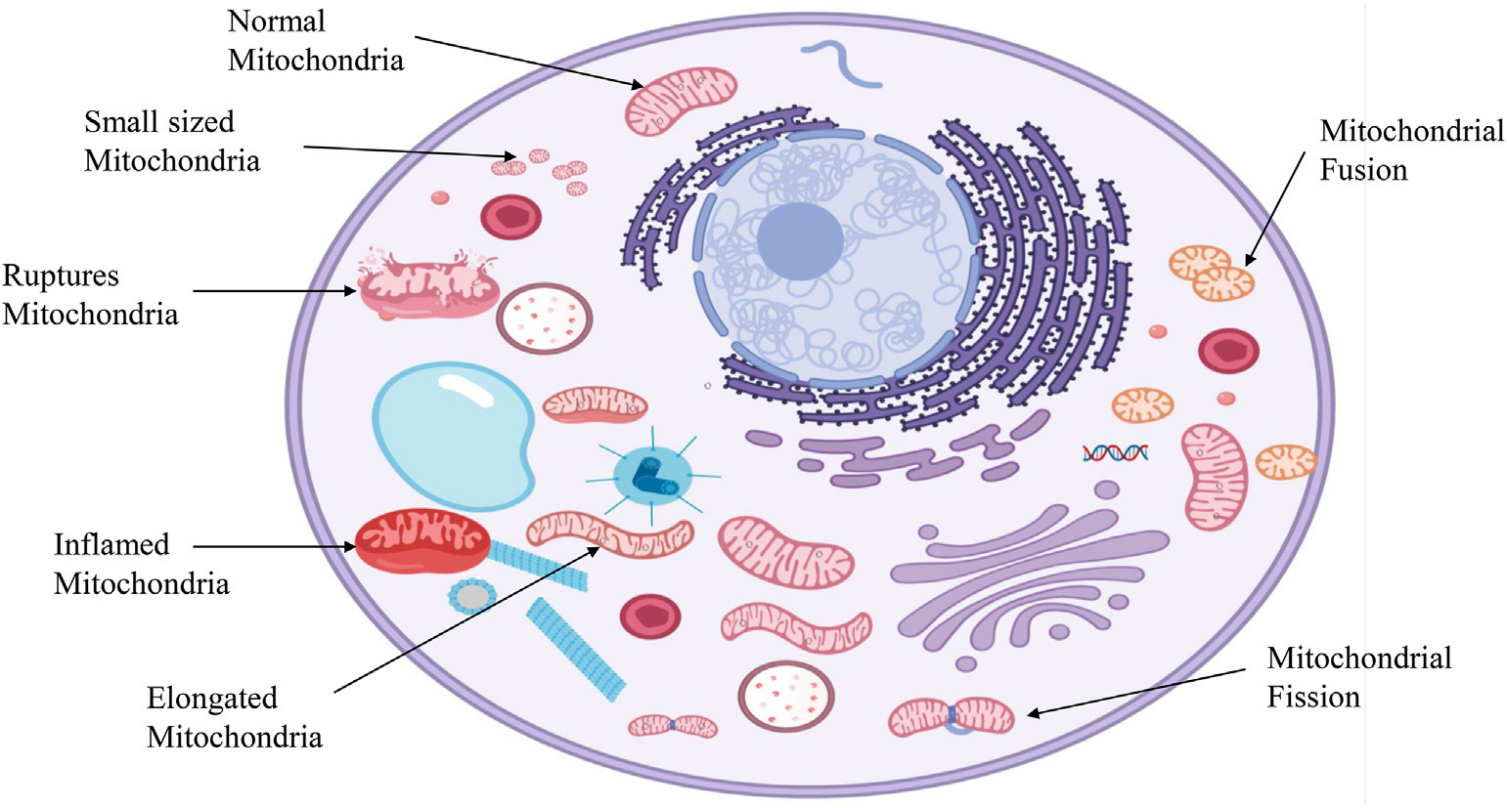
**Mitochondrial abnormalities leading to altered bioenergetics and disease pathology.** Abnormalities in mitochondria can lead to abnormal bioenergetics followed by disease. An imbalance between mitochondrial fusion and fission can cause detrimental bioenergetics. Inflammation of mitochondria due to osmotic imbalance between the matrix and cytosol may affect ATP production and lead to various diseases.

**Fig. 3:**
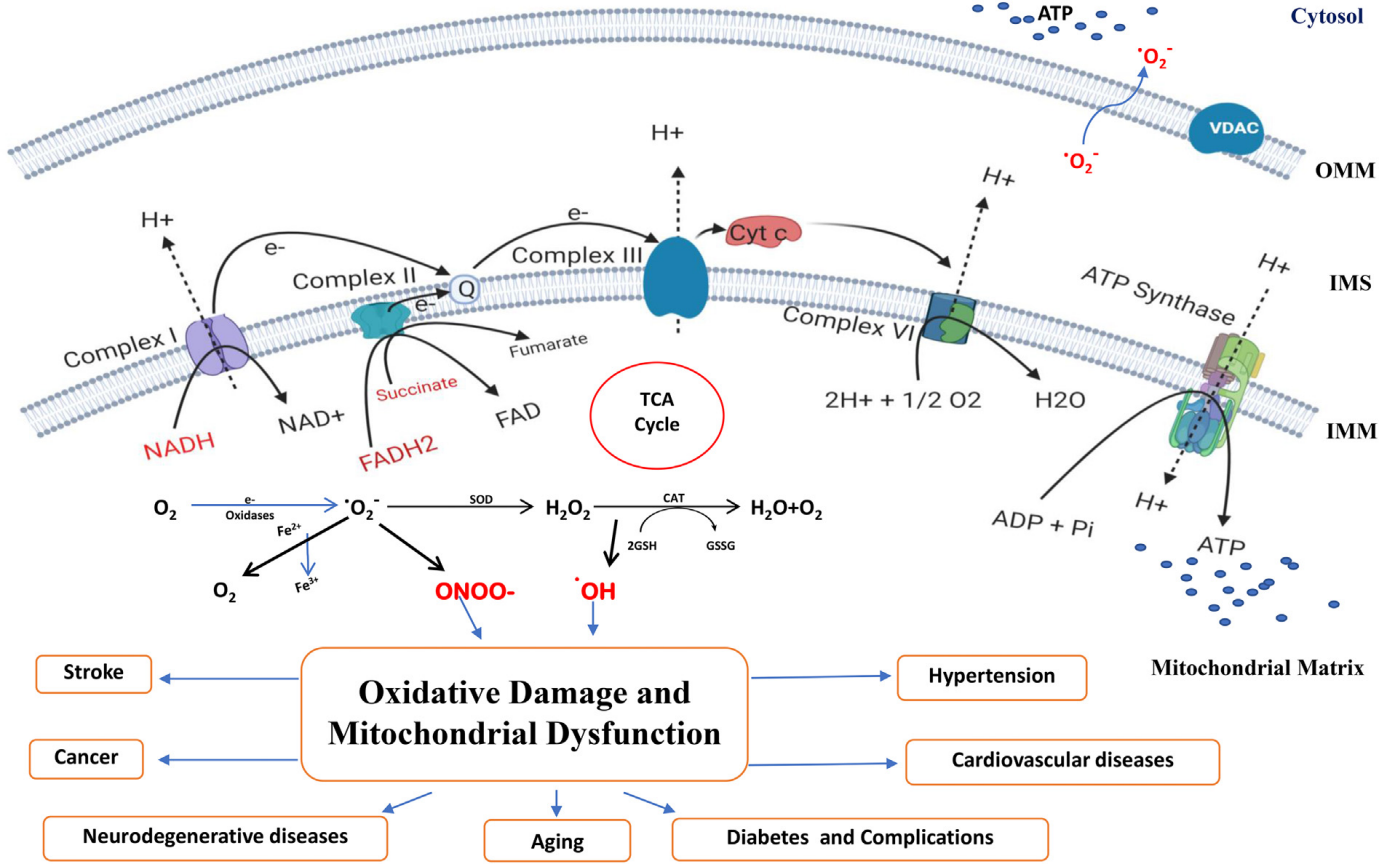
**Mitochondria as a source of ATP production and reactive oxygen species (ROS).** The figure shows the electron transport chain present in the inner mitochondrial membrane and the process of ATP synthesis and production of ROS as a byproduct. The complexes (I-IV) transfer electrons and reach oxygen, the final electron acceptor. This flow of electrons also pumps protons out of the matrix concomitantly, creating an electrochemical gradient that helps synthesise ATP from ADP and inorganic phosphate (Pi). The ROS such as ONOO- and ^•^OH generated due to leak of electrons can lead to oxidative damage and mitochondrial dysfunction, which play a central role in the pathogenesis of many diseases.

Oxidative phosphorylation (OXPHOS), which occurs in the inner mitochondrial membrane, is responsible for generating ATP in mitochondria. The enzymatic reactions involved in Kreb's cycle resulted in the formation of NADH and FADH_2_, two reducing equivalents that are requisite for the transfer of electrons into the respiratory chain [6]. Fig. 2 shows the mitochondrial abnormalities that lead to altered bioenergetics and functions in the cell. The invention of the electron transport chain and the initial oxidative phosphorylation studies illustrate mitochondrial critical role in the cellular energy process [7]. Bioenergetics refers to the molecular physiology and chemistry of energy metabolism. After the findings of various leading scientists, including Ernster, Lenhinger, Boyer, etc. Mitochondrial bioenergetics gained importance during the late 1940s and 1950s. Mitochondrial bioenergetics is considered an important measure to assess the pathogenesis of mitochondrial diseases. Various oxygen-related free radicals are produced as the byproduct of aerobic respiration, including superoxide, hydrogen peroxide and hydroxyl radicals. Fig. 3 shows the process of generation of free radicals and ATP production in mitochondria. The production of mitochondrial ROS occurs due to inefficiencies in the ETC that cause most of the macromolecular disruption but under controlled production, which play significant roles in cell signalling [8].

In the electron transport chain process, four multimeric complexes and an ATP synthase also known as complex V are found in the inner mitochondrial membrane. These complexes are involved in production of ATP via OXPHOS process. Complex I or NADH dehydrogenase is a L-shaped structure with 45 subunits, it is the first and most complicated macromolecular complex of the whole mitochondrial ETC. Interestingly, it is the largest complex with a molecular mass of 1000 kDa. Complex I consist of iron-sulfur clusters (Fe–S), flavin mononucleotide (FMN) and a lipid-soluble electron carrier (CoQ) which is implanted in the lipid bilayer of the inner membrane of mitochondria. The NADH produced in the TCA cycle is oxidized to NAD^+^ with the help of this holoenzyme, and the released electrons are further donated to FMN, which further reduced to FMNH_2_ [9]. The released electrons are further passed to CoQ via a series of Fe–S clusters.

Moreover, four protons are pumped from the mitochondrial matrix to the inner mitochondrial membrane as the electrons pass through the Fe–S clusters. The CoQ is reduced to CoQH_2_ by accepting two protons from the mitochondrial matrix. However, the more precise crystal structure of complex I is not available. Nevertheless, the functional system provides the knowledge of the fundamental mechanism of electron transfer and proton pump. The function of complex I is to transfer the electrons from TCA-derived NADH to UbQ that further shuttles them onto complex III.

Moreover, complex I is the major hotspot of superoxide formation in the ETC. The formation of superoxide occurs either by inhibition of complex I or retrograde signaling pathways in the electron transport chain. Retrograde signalling is generally the regulation of nuclear gene expression in response to functional alterations in organelles [10]. It is a pathway of communication from mitochondria to the nucleus under normal and pathophysiological conditions. It involves some regulatory factors that can sense and transmit the mitochondrial signals to effect changes in nuclear gene expression accordingly to accommodate cells to defects in mitochondria [11]. It is documented that 60 classes of compounds can inhibit complex I via overlapping docking sites within UbQ binding region [12].

### Reverse Electron Transport in mitochondria

Reverse Electron Transport (RET) refers to the reversibility of the Complex-I reaction of the mitochondrial electron transport chain. While in the forward reaction by Complex-I, electrons from NADH are removed and transferred to ubiquinone. In reverse flow, a few electrons from reduced ubiquinol are driven upstream by the high membrane potential to Complex-I. High proton motive force and high ratio of ubiquinol to ubiquinone [13,14]. In RET, the energy consumption is about five times greater than the released energy during the forward reaction, along with a higher rate of ROS production. Recent studies and evidence have shown that RET may occur in vivo and the role of RET generated-ROS in signaling in physiological and pathological conditions and in regulation/extension of lifespan [15,16]. Scialò et al. [15] showed that increased ROS production via RET mechanism from reduced ubiquinone could help extend lifespan in fruit flies by maintaining Complex-I. If conserved in mammals, this strategy can be a novel therapeutic approach for age-related diseases.

Complex II is also known as succinate dehydrogenase (SDH), is the smallest macromolecular complex of the ETC. Complex II comprises of four subunits of proteins: flavoprotein (SDHA), Fe–S subunit (SDHB), and succinate dehydrogenase subunits C and D (SDHC and SDHD) [17]. However, compared with complex I, III and IV, complex II does not support inner mitochondrial membrane proton gradient as it does not involve proton pumping. Moreover, complex II is not considered a part of traditional electron flow as from complex I and complex III; complex II creates a supplementary pathway from TCA cycle to ETC, where the electron released by the complex II helps maintain the potential of mitochondrial membrane [18]. In complex II oxidation of succinate to fumarate and reduction of FAD to FADH_2_ takes place. Furthermore, two electrons are released to a series of Fe–S clusters, which ultimately leads to CoQ when FADH_2_ is oxidised back to FAD. However, these series of reactions do not help directly in ATP generation. The two electrons produced from these reactions are then transferred to membrane-bound CoQ. Additionally, CoQ is reduced to CoQH_2,_ which acts as a transporter of an electron to complex III and complex IV [19].

Complex III, also known as *cytochrome b*_1_ complex, is well documented in the literature. It consists of 11 protein subunits, out of which nuclear DNA encodes ten and one is encoded by the mitochondrial genome. Moreover, complex III is composed of two CoQ binding sites, a Fe–S Rieske protein, *cytochrome c*1 (one heme group) and *cytochrome b* (two heme group) [20]. Moreover, complex III is very well known for superoxide formation due to electron leakage. It is caused either by an imbalance in mitochondrial membrane potential or by exploiting UbQ-binding sites. An event named Q cycle takes place in complex III in which the two binding sites of UbQ takes part, and in which oxidation of two molecules of UbQ takes place at Q_O_ site, and reduction of one molecule of ubiquinone takes place at QI site, which helps complex III for proton transfer across the mitochondrial inner membrane. Complex I and complex II generates CoQH2, which handovers two electrons to complex III, out of which one electron is transferred to Fe–S Rieske protein, *cytochrome c and c*1, which moves to complex I [21]. The second electron is transferred to cytochrome *b*, and CoQ is converted to CoQ·. Another CoQH_2_ attaches itself to complex III, and it handovers a second pair of electrons via the same process. The main difference is that CoQH_2_ is generated as CoQ from the above reaction accepts an electron. Although mitochondrial matrix pumps four protons to intermembrane space in the whole process, two molecules of CoQH_2_ are oxidised to two molecules of CoQ, whereas CoQ is reduced to CoQH_2_ [22].

Complex IV, also known as *cytochrome c* oxidase, is composed of 13 subunits in mammals, out of which nuclear DNA codes 10 subunits and 3 subunits are coded by mitochondrial genome [23]. It also consists of *cytochrome a* (heme group), *cytochrome a*3 and two metallic centres (Cua and Cub). Initially, when complex III diffuses two reduced *cytochrome c* proteins, they join complex IV, and donates two electrons to CuA centre and then they further transferred to *cytochrome a* [23]. Shared electrons are used to reduce *cytochrome a*3 and Cub. As *cytochrome a*3 and Cub are in reduced form, a peroxide bridge can be formed between them as an oxygen molecule can bind to a pair of electrons. Generally, in this process, oxidation of four reduced *cytochrome c* takes place, and two H_2_O molecules are produced with the help of one oxygen and four protons present in the matrix [24]. Four protons are also transferred to the intermembrane space of mitochondria from the mitochondrial matrix.

ATP synthase is an enzyme complex that is present in the inner mitochondrial membrane; the function of this complex is to generate adenosine triphosphate (ATP) by linking the inorganic phosphate to ADP and converting the energy of the proton gradient created by the whole electron transport chain. ATP synthase consists of an F0 domain that is present in the inner mitochondrial membrane) and F1 domain in the mitochondrial matrix. The proton flux is governed by F0 domain by the membrane which creates torque, which creates a circular movement of F1 domain which results in the generation of 3 ATP molecules upon one turn of the enzyme [25]. Several studies demonstrated the inhibition of these complexes of electron transport chain in different pathological conditions. Table 1 summarizes the inhibitors of all the respiratory complexes which can be targeted as a therapeutic strategy in various diseases.

**Table 1:** Toxins and inhibitors of mitochondrial respiratory complexes.

Complexes	Toxins/Inhibitors	Mechanism of Action	References
Complex-I	Rotenone	Inhibition of electron transport from the iron-sulfur centers in complex I to ubiquinone.	[26]
	Myxothiazol	Inhibits mitochondrial respiration in the bc1 complex of the respiratory chain.	
	Metformin	Directly targeting ETC complex 1, resulting in inhibition of ATP synthesis	[27]
Complex-II	Thenoyltrifluoroacetone (TTFA)	Inhibits complex-II by binding to two ubiquinone binding sites, Qp and Qd.	[28]
	2-[1-Methylhexyl]-4,6-Dinitrophenol	Targets succinate dehydrogenase flavoprotein subunit.	
Complex-III	Antimycins	Prevents transfer of electrons from *cytochrome b* to c_1_	[29]
	Ubiquinone Q2, B-2-Octylglucoside	Targets ubiquinol-*cytochrome c* reductase iron-sulphur subunit	
Complex-IV	Cyanide	Binds with iron and prevent transfer of electrons.	[30]
	Carbon Monoxide	Binds with the reduced form of iron in the heme groups and blocks transfer of electrons to oxygen.	[31]
	Azide	Reacts with ferric form of Complex-IV and blocks further electron flow.	[32]
Complex-V	Efrapeptin	F1-targeting, binding pocket localized in α, β and γ subunits	[33]
	Oligomycin	Binds to the Fo subunit and inhibits the apoptosis induced by Bax	
	Aurovertin B	Localized mainly in the β subunit.	

## Alterations in mitochondrial bioenergetics

Metabolic imbalance of energy production, the input of nutrient signals, or/and oxidative respiration induces ‘mitochondrial dysfunction’. Mitochondrial dysfunction is typically described as a lack of ATP production and maintenance in mitochondria. In addition, the term is intended to describe ill-fit physiology for mitochondrial patients suffering from several disorders such as substrate catabolism, calcium buffering, transport of iron, mitochondrial DNA mutations, mitochondrial dynamic alterations, morphology, size, ROS generation and apoptosis. The main sites of ROS generation in ETC are the complexes I and III, where the electrons may leak and get accepted by oxygen to form a superoxide ion (O_2_^•–^), the chief ROS in mitochondria. However, superoxide is comparatively less reactive, but it can form peroxynitrite (ONOO^−^) by rapidly reacting with nitric oxide (NO) and other reactive nitrogen species (RNS) such as nitrogen dioxide (NO_2_) and dinitrogen trioxide (N_2_O_3_). Most superoxide gets converted into hydrogen peroxide (H_2_O_2_) either by natural dismutation or enzymatic dismutation by SOD (superoxide dismutase). H_2_O_2_ has the ability to oxidize the thiol groups of some enzymes, which causes their inactivation.

Highly reactive hydroxyl radical (^•^OH) can be produced by H_2_O_2_ via Fenton reaction [34], which is very dangerous to an organism. Under normal physiological conditions, a balance is maintained between free radicals or ROS production and antioxidant defense mechanisms in the living systems. Antioxidants can be generated naturally in the body (endogenous), such as catalase (CAT), SOD, glutathione (GSH), and Coenzyme Q10 (CoQ10), or can be supplied through foods or supplements (exogenous), for example, vitamins A, C and E. However, an imbalance between these prooxidants and the antioxidants in a living system ultimately leads to oxidative stress associated with tissue and cellular damage [35]. Oxidative damage to internal mitochondrial membrane lipids (lipid peroxidation) such as cardiolipin (very sensitive to ROS/RNS) leads to increased leakage of protons and ions in the matrix and a loss of electrochemical gradient, resulting in the loss of function of ETC or OXPHOS, for example, the activity of the complexes gets deteriorated. The oxidative stress in mitochondria also causes the *cytochrome c* to get released into the cytoplasm, resulting in apoptosis. Since mtDNA is situated in proximity to ROS production sites. Also, it lacks the protective histones, it is more vulnerable to DNA defects or damage [36], and the oxidative damage to mtDNA results in single or double-stranded DNA breaks; the mitochondrion's inability to repair the damaged mtDNA results in its segregation into the mitochondrion zone, which will be targeted for mitophagy after the fission. There is a link between mitochondrial dynamics and bioenergetics; mitochondrial fusion is linked to increased production of ATP, whereas inhibition of this process or fission is associated with impaired OXPHOS as well as excessive generation of ROS. The equilibrium between fission and fusion is controlled by alterations in the supply of nutrients and metabolic demands, leading to mitochondria adaptation under different conditions. Overall, when the ROS is produced at extreme levels, it leads to a decline in the physiology of cells [37]. Table 2 shows the mitochondrial alterations associated with the pathophysiology of several diseases.

**Table 2:** Mitochondrial abnormalities associated with disease pathology.

Disease	Mitochondrial abnormality	Reference
Alzheimer's disease	• Aggregation and accumulation of Aβ in mitochondria of AD brain • Impairment of TCA enzymes • Impairment of ETC Complexes I, III, IV	[38]
Parkinson's disease	• Increase in ROS and oxidative stress • Decrease in mitochondrial Ca^2+^ • Impairment of ETC Complex I	[39,40]
Huntington's disease	• Impairment of ETC CII, III, IV • Defect in mitochondrial Ca^2+^ handling • Oxidative dysregulation	[41]
Diabetes	• Increased ROS production • Impairment of mitochondrial biogenesis • Decreased β-oxidation and ATP production	[42,43]
Hypertension	• Decreased activity of complex IV • Increased NADPH oxidase activity • Vascular oxidative stress	[[44], [45], [46]]
Cardiovascular disease	• Impairment of complex I and IV • Production of excessive ROS • Reduced ATP production • Mitochondrial membrane rupture and matrix scarcity	[[47], [48], [49]]
Stroke	• Impairment in calcium influx • Impairment across mitochondrial membrane gradient which leads to dysfunction in ETC • Low ATP synthesis • Cytochrome *c* dysfunction	[[50], [51], [52]]

## Neurodegenerative diseases

Neurodegenerative disorders are different kinds of disorders that are described by progressively selective loss of anatomically or physiologically related neuronal frameworks, for instance, Alzheimer's disease (AD), Parkinson's disease (PD), lateral amyotrophy (ALS), and Huntington's disease (HD). We will focus on mitochondrial dysfunction, especially the altered bioenergetics, such as oxidative stress and mutations in genes involved in the pathogenesis of these diseases. Mitochondria, which are crucial in ageing, are the key controllers of cell survival and death. They have been related to many specific proteins in the hereditary types of neurodegenerative disease. Since neurons are highly dependent on OXPHOS for their considerable energy demands and contain fewer antioxidants than other cells, they are highly susceptible to oxidative stress. Besides, for certain neurodegenerative disorders, the defects in the ETC Complex-I role have been included, which hinders ATP formation and produces ROS that can harm mitochondria extraordinarily [53]. Unexpectedly, the deficiency in numerous ETC complexes is related to different neurodegenerative problems: PD Complex I, HD Complex II, and AD Complex IV. An investigation directed by Cottrell et al. showed that complex-IV deficiency additionally existed in locales of the CNS, which is generally influenced by neurodegeneration. It was proposed that neurons with a shortage of complex IV were bound to be lost in areas with higher odds of OXPHOS dysfunction. High degrees of Complex IV-lacking neurons, which means decreased cytochrome *c* oxidase activity, appeared to be profoundly present in AD when contrasted with the normal brain. This Complex IV insufficiency could be brought about by mutation in mtDNA, bringing about oxidative damage [54]. Another study done by Tabrizi et al. demonstrated significantly reduced activity of Complex-II and III in the HD brains. On account of nitric oxides, the hindrance of Aconitase had been extended most significantly, showing the frequency of complex-II and complex-III inhibitions, in this way giving an extra procedure for the production of ROS [55].

### Alzheimer's disease

Alzheimer's disease or AD is the most common form of neurodegeneration. Common characteristics of AD include intracellular neurofibrillary tangles (NFT) and senile plaques composed of β-amyloid (Aβ) peptides (formed due to proteolytic cleavage of the AβPP or APP (amyloid-β or amyloid precursor protein) via β- and γ-secretases). The postmortem of the brain of an AD patient in early studies revealed lower or abnormal activity of three enzymes involved in TCA: pyruvate dehydrogenase, isocitrate dehydrogenase, and α-ketoglutarate dehydrogenase; as well as of ETC complexes I, III, and IV in AD patient's lymphocytes and platelets. This impaired bioenergetics leads to ROS production, oxidative damage (protein and DNA), lipid peroxidation, and decreased ATP synthesis, and these are said to be early events in AD [56]. ATP's anterograde and retrograde mechanisms are supplied to neurons by mitochondria get disturbed because of mitochondrial defects [57].

The link between mtDNA mutations and mitochondrial dysfunction in AD was investigated by Coskun et al. [58] and it was found that the mtDNA defects were more in AD brains and healthy aged brains than in young and healthy brains. Thus, it was concluded that mutated mtDNA accumulation is age-related in AD pathogenesis. This accumulation prompts ROS generation in neurons, activating β- and γ-secretases, which adds to APP level. A gene expression analysis by Reddy et al. concluded that “mitochondrial energy metabolism is disrupted by the expression of mutant APP and/or Aβ" after observing the abnormal expression of mitochondrial-encoded genes in Tg2576 mice. Several cellular, molecular and animal-model studies have shown that mutant APP or Aβ enters mitochondria in neurons. Then there is an elevation of Aβ levels, clearly resulting in increased plaques and ultimately impairment of ETC and the generation of free radicals [59]. An investigation in N2a cells observed increased expression of mitochondrial-encoded genes in ETC complexes I, III, IV, and V in the cells treated with Aβ compared to the N2a cells untreated with Aβ. Thus, learning disabilities and mitochondrial dysfunction in AD brains are related to Aβ accumulation in mitochondria.

It has also been reported that greater levels of Aβ and ultimately overproduction of mitochondrial ROS impair the mitochondrial dynamics, that is, fission and fusion affecting the morphology and distribution of mitochondria. Some studies corroborated the link between ROS and abnormal dynamics. Barsoum et al. [60] observed that mitochondria treated with Aβ experience fission in AD. In contrast, Yoon et al. [61] observed the fragmentation of mitochondria and increased ROS in cells exposed to high glucose concentrations. Bernard et al. [62] also observed mitochondrial fragmentation in cells treated with rotenone due to increased ROS and decreased ATP production. These observations suggest that increased ROS in AD may alter mitochondrial dynamics and damage neurons. Other studies supported the decrease in overall mitochondrial dynamics associated with increased APP and Aβ. In a confocal and electron microscopic analysis by Wang et al. [63], a study on M17 cells transfected with wild-type or mutant APP was carried out. It was found that approximately 40% of the cells with over-expressed with wild type APP (APPwt) and more than 80% of these cells over-expressed with mutant APP (APPswe) showed disturbed mitochondrial dynamics, particularly fragmentation; also that increase in Fis1 level is necessary for fission in both types of cells.

### Parkinson's disease

The second most widely recognized neurodegenerative disease is Parkinson's disease (PD), which involves a loss of dopaminergic (DA) neurons in substantia nigra pars compacta (SNpc) as well as the occurrence of Lewy bodies which are made up of a group of α-synuclein (α-Syn) protein, and this disease is often linked to ageing and mitochondrial dysfunction. Several studies have revealed and substantiated the role of mitochondrial dysfunction in PD, including the defects in ETC or bioenergetic defects, which supports the aggregation of α-Syn. It was first demonstrated in 1983 that mitochondria are involved in PD pathology when an unanticipated event involving intake of MPTP (1-methyl-1, 4-phenyl1, 2, 3, 6-tetrahydropyridine), a toxin that caused PD-like symptoms in youthful drug addicts. MPP^+^ (1-methyl-4-phenylpyridinium ion), the metabolite formed from MPTP targets the Complex-I of ETC and blocks the transfer of electrons, impairing the OXPHOS process and increasing production of ROS, thus inducing oxidative stress [64]. Another drug, rotenone, works similarly; however, unlike MPP^+^, it is not specific to dopaminergic neurons. Several other toxins are recognized to date to inhibit complex-I according to the exposure level, such as 1-trichloromethyl-1, 2, 3, 4-tetrahydro-β-carboline (TaClo), pyridaben, chlorpyrifos and fenpyroximate. Examination of the postmortem PD patients discovered that different brain regions other than frontal cortex [65], putamen, hippocampus, and pedunculopontine nucleus show deficiencies in Complex I activity, causing mitochondrial dysfunction. In PD lymphocytes and platelets, deficiencies of other complexes have also been outlined. However, all the defects predominantly occur in the neurons of SNpc, and the appropriate reason behind this remains obscure [66]. Mitochondrial dysfunction in PD has also been linked to genetic mutations, which generally accumulate with time or age, and several studies have been performed to describe it. Genes like PRKN (Parkin) and PINK1 (PTEN-induced putative kinase; PARK6) and their mutations are involved in an autosomal recessive form of familial PD, whereas mutations in genes like SNCA (Synuclein Alpha) and LRRK2 (leucine-rich repeat kinase 2) are associated with the autosomal dominant form of familial PD. Parkin deficiency in mice has shown decreased complexes I and IV activity, reduced mitochondrial respiration with higher oxidative stress, and increased sensitivity to rotenone. Moreover, lack of PINK1 also results in decreased mitochondrial respiration, increased susceptibility to oxidative stress, reduction in membrane potential and various morphological defects in mitochondria [67]. Overexpression of PINK1 helps in reinstatement of mitochondrial morphology, inhibiting both ROS formation and release of *cytochrome c*, thus preventing apoptosis of neurons. It has been shown in studies that after reducing the mitochondrial membrane potential, the depolarized mitochondria lead to the accumulation of PINK1 on the outer membrane of mitochondria and it has to recruit parkin in order to carry out mitophagy. In Drosophila studies, the knockout of both the genes revealed that they function in the same pathway and together in mitophagy [68]. Thus, mutations in both these genes cause mitochondrial dysfunction and inhibition of mitophagy. SNCA encodes for the protein α-synuclein, and changes in the SNCA gene instigate abnormal α-synuclein forms to aggregate, which have been a part of Lewy bodies [69]. α-Syn and mitochondrial import and aggregation impede complex I activity and cause neurodegeneration in dopaminergic neurons. Research by Devi et al. [70] found that the accumulation of α-syn caused dysfunction of Complex-I, and an unevenness had been developed between the amount of ROS and level of antioxidants. In PD, PINK1 downregulation has consequences for offsetting mitochondrial splitting (fission and fusion). It has seemed in an experiment led by Rojas-Charry et al. [71], that fusion was supported when PINK1 was over-expressed, as showed by a decreased presence of divided mitochondria as compared to PINK1 control cells. Drp1 and Mfn2 levels in PINK1-missing cells were lower when contrasted with PINK1 control cells. In correlation, the expression of Fis1 expanded essentially in PINK1-missing cells instead of cells carrying the gene. Fis1 expression is elevated because of aggregation of calcium and changes in the membrane potential, forestalling electrochemical gradient development, and empowering overproduction of ROS. In this manner, PINK is pivotal for neurons since its nonappearance causes a generous reduction in Mfn2 and an amplification in Fis1, encouraging fission instead of fusion. Ageing and mutations in PINK1 would cause dysfunction in the mitochondrial apoptotic pathway [72]. Astrocytes inadequate in PINK1 showed impaired cell development, conceivably because of abnormal apoptosis [72].

### Huntington's disease

Huntington's disease or HD is an autosomal dominant neurological disease caused due to mutations in the Huntingtin (htt) gene that expands CAG repeats. A large body of literature concludes that mitochondrial energetics and metabolism are disrupted in HD, and it is supported by many studies in mutant htt transgenic mice, cell culture models and HD patients. The activity of ETC complexes II, III and IV is reduced in HD brains [73]. Mitochondrial inhibitors such as 3-nitropropionic acid that generally inhibits complex II can stimulate striatal degeneration and movement disorder in rodents and primates; however, when subunits of complex II are over-expressed, reduction in cell death in striatal neurons with mutant htt is observed [74]. In a spectrophotometric analysis, impaired activities of the OXPHOS enzymes were observed in basal ganglia of HD brain. In contrast, in frontal and parietal cortex and cerebellum, no impairment in the enzymes was found [75]. Apart from complex II, defect in complex III activity in the putamen and caudate regions and in complex IV in putamen has been detected.

## Metabolic disorders

The metabolic syndrome (MetS) is a significant group of pathological conditions such as insulin resistance, obesity, diabetes, hypertension, cardiovascular diseases, etc. The individual risk factors for cardiovascular disease are the elements of metabolic syndrome. Emerging evidence revealed the association of mitochondrial abnormalities in several health conditions, including type-2 diabetes, obesity, cancer and ageing-associated neurodegenerative disorders.

### Insulin resistance and diabetes

Insulin resistance is an early characteristic of type-2 diabetes. Type-2 diabetes mellitus (T2DM) has been accounted for with oxidation impedance, diminished mitochondrial content, hereditary factors, impaired and diminished insulin discharge from beta cells, lower rates of oxidative phosphorylation, and the advancement of unreasonable ROS [76]. Insulin obstruction is an element of type 2 diabetes, and parts of the cardiometabolic condition, including hypertension and dyslipidemia, which together add to an extensive hazard for cardiovascular disease. The job of mitochondrial dysfunction in the development of T2DM is progressively clear, particularly with the movement of diabetic entanglements including retinopathy, nephropathy, neuropathy, and cardiovascular diseases [77,78]. One of the characteristics of type-2 diabetes is insulin resistance which is linked to mitochondrial dysfunction.

Moreover, it has been seen in skeletal muscles of patients of insulin resistance that there are some alterations in mitochondrial function and increased lipid peroxidation levels. Additionally, in type-2 diabetes, genes of OXPHOS and mitochondrial biogenesis have been downregulated. Studies have shown that insulin signalling is hindered in insulin-resistant patients, with high levels of serine phosphorylation of IRS1, low levels of AKT phosphorylation, and reduced translocation of GLUT4 to the sarcolemma membrane, hence altering in glucose uptake. Above mentioned defects are also pathway-specific, with conserved activity of the ERK-MAPK pathway in spite defects in AKT pathway [79]. Besides skeletal muscles, liver is another organ which is affected by insulin resistance, as it results in failure to suppress gluconeogenesis, but encouraging hepatic glucose production. AKT and FOXO mechanisms are involved in insulin resistance which inhibits the transcription of two gluconeogenic enzymes, phosphoenolpyruvate carboxykinase (PEPCK) and glucose-6 phosphatase (G6Pase). Hepatic steatosis and non-alcoholic fatty liver disease are also caused by insulin resistance, which increases lipogenesis.

Further, insulin signaling is reduced due to lipid accumulation through activation of PKC-ε and JNK1-dependent pathways [80]. Tumor necrosis factor-α in hepatic tissue activates the ROS and further regulates kinase 1 via apoptosis signaling pathway. Shreds of evidences suggest that alterations such as point mutations can prompt the progression to diabetes. Both mitochondrial genome and nuclear genome has the ability to encode mitochondrial proteins, however there are some evidence suggests that mtDNA alterations in nuclear-encoded proteins such as PGC-1a and NDUFB6 which are linked to both T2DM and insulin resistance [81]. Likewise, pancreatic beta cell breakdown is linked to mitochondrial dysfunction. It was exhibited in an investigation that due to excessive ROS generation in β cells, it was linked to the failure of favoring low levels of insulin secretion by beta cells. It was also established that mitochondrial functions control the insulin secretion by β cells because mitochondria produced the metabolites linked with insulin secretion, like the ATP generated, which couples the blood glucose level with insulin secretion. When blood glucose enters the β-cell, high ATP to ADP ratio depolarizes the plasma membrane by closing the ATP- sensitive K channel leading to an increased calcium influx encouraging the fusion of insulin-containing granules with the plasma membrane, consequently resulting in insulin secretion [82,83]. Any defect in this process can lead to a disruption in insulin secretion and cause T2DM. Mitochondria are a well-known source of energy production which is required for normal functioning of cells and they also play an important role for β cells in insulin secretion [84].

### Hypertension

Hypertension is one of the significant hazard determinants of metabolic syndrome. Many hazard factors related to hypertension incorporate obesity/overweight, smoking, diabetes, inactive way of life, absence of physical movement, significant levels of salt, liquor consumption, insufficient calcium, potassium or magnesium intake, lack of nutrient D, stress, ageing, chronic kidney disease, and adrenal and thyroid issues or tumours as well as the genetic element. Hypertension is related to modifications in the biogenesis and other aspects of mitochondria. Note that heart function is subject to mitochondrial activity [85]. The cardiomyocytes are the cells with high mitochondrial thickness because they need a vast and consistent supply of ATP to support their repetitive contraction and the usefulness of different ion transporters. On this point, hypertension-related cardiovascular hypertrophy is ascribed to be changed in the utilization of metabolic substrates, dysfunction of the ETC and ATP synthesis [86]. Because of the high amount of mitochondrial material of cardiovascular cells, various obsessive heart issues have been related to adjustments in mitochondrial fission and fusion procedures. In a study conducted on rodents, it was seen that there was a low level of mRNA fusion proteins MFN1 and MFN2, and OPA1, which suggests that in hypertension, there are high chances of mitochondrial fragmentation. The treatment of refined neonatal rodent cardiomyocytes can be done with norepinephrine advances as mitochondrial fission is related to lowering the mean volume of mitochondrial and increasing the total number of mitochondria per cell.

Moreover, it is also seen that norepinephrine mediates the increase of cytoplasmic Ca^2+^ that relocates calcineurin advances to mitochondrial fission protein DRP1 [87]. The movement of cytosolic DRP1 towards mitochondria during fragmentation is an organized procedure, including post-transcriptional changes of DRP1. The relationship between mitochondrial fission and the two ROS advancement, just as cell apoptosis can be another critical commitment of mitochondrial fission actuated by norepinephrine. In such a manner, it is notable that *cytochrome c* is discharged at DRP1-mediated mitochondrial fission sites employing Bax-lined pores, which trigger cell apoptosis. Curiously, the two ROS development and myocardial cell apoptosis are broadly proposed in hypertension-related left ventricle hypertrophy as components associated with the genesis and movement of the disease. In addition, upgrades in the energetic mitochondrial metabolism, including diminished respiration and ATP creation, are additionally trailed by hypertension-incited mitochondrial changes. It is proposed in such a manner that while fusion advances the respiratory limit, mitochondrial fission is related to a decreased oxidative metabolism [85].

### Cardiovascular disease

Cardiovascular diseases (CVD) are the primary cause of mortality worldwide, with complex aetiology involving various risk factors. In cells, different metabolic imperfections, unreasonable reactive species (ROS) generation, vitality deficiency, autophagy deregulation, endoplasmic reticulum (ER) stress, and apoptosis initiation lead to pathogenesis of CVD. The mitochondrial dysfunction assumes a pivotal job in such cell perturbation. In addition, mitochondria can control cell stress reaction by utilizing retrograde signaling while denying the mitochondrial film likely outcomes in the initiation of a few flagging proteins, which directs many pressure-responsive qualities nucleus. Practical imperfections of cardiovascular mitochondria in CVD lead to expanded oxidative stress, diminished advancement of ATP and vitality, expanded apoptosis of cells, and impaired autophagic mechanism [48].

The mitochondrial respiratory chain is the main pathway for generating ATP molecules in the cell. The respiratory chain uses more than 98 per cent of electron transport for ATP synthesis to operate effectively under normal conditions. ROS's controlled development is instrumental in inducing chemical preconditioning of protective signaling mechanisms [88]. Uncoupling the mitochondrial ETC from ATP creation adds to ROS overproduction, bringing about oxidation of lipids and proteins and severe damage to the cells. Improvement of ROS advances atherogenesis at all phases by initiating endothelial dysfunction, tube inflammation, gathering of low-thickness oxidized lipoprotein (oxLDL) in the arterial wall, development in the propelled plaque with conceivable movement to plaque burst. Be that as it may, the heart generally creates low ATP levels because of the substantial ATP admission and fast ATP turnover [89]. Mitochondrial creatinine kinase controls the respiratory chain structure and advances the combination of the high-vitality phosphate. Phosphocreatine delivered from this creatine catalyst assumes a significant job in keeping up myocardial ATP cushion content. Moreover, the compound happens in two structures: profoundly receptive octameric and dimeric less dynamic in powerful balance. For coronary illness, the harmony between the two structures is adjusted toward the dimer because of expanded octamer separation and the development of latent crystalloids from octameric creatine kinase. The cardiovascular breakdown has debilitated OXPHOS contrasted with ordinary and diminished ATP levels bringing about decreased cardiac output [90].

### Stroke

An ischemic occasion happens when the bloodstream to the brain tissue given by impeded arteries is diminished. The loss of oxygen and supplements adds to upset cell homeostasis and, at long last, cell demise. A critical reduction of the central cerebral bloodstream in a patient with an ischemic stroke adds to the loss of glucose and oxygen, which causes brain damage. One of the contributing components is oxidative damage, which builds the danger of ischemic cerebrum injury. ROS is developed under various conditions from various living cells, including hypoxia, cerebral ischemia, cytokine incitement, and serum exhaustion, with the fundamental ones being mitochondria, 5-lipoxygenase, and NADPH oxidase [47]. Mitochondria are the primary wellspring of intracellular ROS. With oxidative stress, free electrons in the mitochondrial ETC can spill out and partner with molecular oxygen, making superoxide anion (O2^•−^) a metabolic side-effect of respiration. The exceptionally dynamic O2 ties to the nitrogen oxide (NO)- forming peroxynitrite anion (NO3), which adequately prompts the advancement of cytotoxic hydroxyl radicals and changes in DNA, protein, and lipid structures. Unreasonable NO development is intervened by different isoforms of the activated nitric oxide synthase (NOS) post-ischemic stroke, including neuronal, endothelial, and inducible NOSs [91]. Damaged macromolecules by ROS and responsive nitrogen species (RNS) assumes a critical job in different physiological and obsessive conditions, including malignant growth, neurodegenerative diseases, and ischemia-reperfusion injury. Holding low ROS levels for typical cell function is significant while expanding mitochondrial action represents an inherent danger of higher ROS. The harmony between ROS generation and clearance is impaired following cerebral ischemia, bringing about oxidative-stress-prompted signaling and cell injury.

## Therapeutic compounds targeting mitochondria

As the knowledge about different aspects of a mitochondrion unravelled over the years, the concept of mitochondrial medicine (targeting particularly mitochondria) has become preferable. The crucial role of mitochondria in regulating a cell's survival and death makes them a potential target for therapeutics of primary pathological conditions, including cancer. The strategies to target mitochondria include inhibition of ETC complexes, uncoupling of OXPHOS, controlling ROS and oxidative stress levels, targeting Kreb's cycle enzymes or specific other proteins of mitochondrial membranes, for example, anti-apoptotic proteins. Here, we will discuss some drugs that target mitochondrial bioenergetics in various diseases and their mode of action. Table 3 shows the mitochondria targetted therapeutic compounds and their mode of action in metabolic and neurodegenerative diseases.

**Table 3:** Therapeutic drugs targeting mitochondria and their mode of action in metabolic and neurodegenerative diseases.

Disease	Drug/Compound	Mode of Action	References
Neurological Disorders	MitoQ	Protect against ROS and lipid peroxidation	[104]
	MitoVit E	Act as ROS scavenger Reduces lipid peroxidation	[105,106]
	Coenzyme Q10	Reduces Aβ peptide accumulation Supports mitochondrial ETC Attenuation of decreased oxidative phosphorylation efficiency and increased H_2_0_2_ production	[[107], [108], [109]]
	MitoApo	Helps in reducing superoxide formation Reduces H_2_O_2_ production Prevents oxidative stress-induced cell death	[110]
	Melatonin	Helps in reducing mitochondria calcium levels Helps in maintain reduced mitochondrial volume	[111,112]
	Nicotinamide adenine dinucleotide	Regulates fission and fusion of mitochondria Restores ATP and NAD^+^ levels Helps in blocking the accumulation of ROS Mitophagy stimulation	[113,114]
	MitoTEMPO	Eliminates mitochondrial superoxide Scavenges ROS Reduces lipid peroxidation and oxidative stress Increases mtDNA fidelity and copy number	[115]
	Szeto-Schiller tetrapeptides	Targeted delivery of antioxidants to the inner mitochondrial membrane Scavenge hydrogen peroxide and peroxynitrite and inhibit lipid peroxidation By reducing mitochondrial ROS, these peptides inhibit mitochondrial permeability transition and cytochromec release, thus preventing oxidant-induced cell death. Rejuvenation of mitochondrial activities in dysfunctional and aging mitochondria	[100,116,117]
Metabolic diseases	MitoQ	Improves arterial stiffening and endothelial function Inhibits ROS generation Protects against inflammation	[118,119]
	Metformin	Decreasing the glucose absorption in the intestine Encourages weight loss in obese patients	[120,121]

## Mitotherapeutics in neurodegenerative diseases

Specific targeting of drugs and compounds to mitochondria faces some difficulties because of impermeability, high negative potential, and bilayer structure of mitochondria. Moreover, for neurological diseases, the blood–brain barrier (BBB) may cause trouble for targeting mitochondria of the brain due to its hydrophilic nature and restrict the therapeutic molecules [92]. Mitochondria-targeted antioxidants, Szeto-Schiller (SS) tetrapeptides, Olesoxime, etc., are therapeutic strategies that can treat neurodegeneration while targeting mitochondria [93].

Mitochondria-targeted antioxidants or MTAs have been discovered, which provide more protection for oxidative damage than the normal untargeted antioxidants, as they can pass easily through the biological membranes of mitochondria, which is the prime site of ROS generation. The untargeted antioxidants are generally conjugated to triphenylphosphonium (TPP), a lipophilic cation (positive charge), for easy delivery into the mitochondrial matrix through the membrane with negative potential [94]. These TPP conjugated antioxidants include: the most studied MitoQ or mitoquinone, which is a derivative of CoQ10, MitoVitE (derivative of tocopherol), MitoLip (derivative of lipoic acid), MitoTempol (SOD mimetic), and MitoPrx (peroxidase mimetic ebselen). Efforts are being made to develop and study the effects of more such antioxidants such as MitoCurcumin (mitochondria-targeted curcumin) and MCAT (mitochondria-targeted catalase) [95].

MitoQ is a compound in which TPP is covalently attached to the ubiquinone moiety of CoQ_10. It_ is considered a neuroprotective agent as it can protect against the common ROS and lipid peroxidation and prevent apoptosis, just like CoQ_10_. It preserves the activity of mitochondria in the absence of glutathione, even in the cells without mtDNA. The position of MitoQ is known to be such that the ubiquinone component penetrates deep into the hydrophobic membrane interior, making it easier to access the core of the membrane, and the TPP ^+^ component exists on the membrane surface. It acts by donating a hydrogen atom from a hydroxyl group of ubiquinol to a lipid peroxyl radical. The ubiquinol can be recycled back to its active reduced form by CII of ETC. The efficacy of MitoQ against neurodegeneration has been corroborated by various in vivo and *in vitro* studies. In a study, MitoQ was observed to inhibit the MPTP-induced changes in behaviour, inactivation of the mitochondrial aconitase, and apoptosis of neurons in PD models [96],. Studies on AD models concluded that MitoQ protects against Aβ-induced toxicity and dysfunction of ETC complexes; prevents cognitive decline, synaptic loss, and astrogliosis [97].

Further, MitoVitE or mitotocopherol is taken up and gets accumulated in mitochondria similar to MitoQ and protects against H_2_O_2_, lipid peroxidation and oxidative stress in tissues, including brain. In fibroblasts of Friedreich's ataxia (FRDA) patients, MitoVitE prevented cell death caused by excessive ROS production [98]. In bovine aortic epithelial cells, it was shown to inhibit (at 1 μM) the release of *cytochrome c*, thus preventing apoptosis while restoring proper bioenergetics. Another study proved to be effective against neurodegeneration in cerebellar granule cells by reducing the accumulation of intracellular oxidants induced by ethanol concentrations [99].

Recent studies demonstrated the protective role of small peptides in various metabolic and neurodegenerative diseases. A small peptide (aromatic-cationic) molecule created by Hazel H. Szeto and Peter W. Schiller were named as Szeto-Schiller or SS peptide. They act as antioxidants that can penetrate and target the inner mitochondrial membrane of several kinds of cells, such as neurons. The most studied of these are SS-20 and SS-31, of which SS-31 is known to protect against or scavenge different ROS, while SS-20 lacks this ability but still can reduce mitochondrial ROS production [100]. A study revealed that in G93A transgenic mice, which represent ALS models, the treatment with SS-31 led to a reduction in oxidative stress markers and neuron cell loss [101]. Recently, Reddy et al. found that SS-31 decreased the mitochondrial dysfunction, Aβ production and helped enhance the mitochondrial biogenesis and synaptic activity in the APP mice model [102]. Both SS-31 and SS-20 prevented the inhibition of ATP production and oxygen consumption induced by MPP^+^ in isolated mitochondria. All these studies support the efficacy of SS peptides for neurodegenerative diseases.

In addition, Olesoxime is a compound having a cholesterol-like structure that targets mitochondria and shows neuroprotective effects. The mechanism of action of this compound involves inhibition of mitochondrial permeability transition pore and decreasing oxidative stress [103].

## Mitotherapeutics in metabolic diseases

A common strategy used for the treatment of metabolic diseases is the use of antioxidants to reduce ROS load. However, the mitochondria-targeted antioxidants have become more common as they accumulate primarily in the mitochondria, where excessive ROS is produced. MitoQ is known to improve endothelial function (by reducing mitochondrial ROS) as well as the arterial stiffening in older adults [118], protects against the onset of hypertension, decreases cardiac hypertrophy in young stroke-prone spontaneously hypertensive rats [122]. It inhibits the high-fat diet (HFD)-induced ROS generation in rat liver tissue and kidneys. The mitochondria-targeted coenzyme Q is also known to be beneficial for mitigating inflammation in metabolic syndrome. For example, it inhibits the activation of NLRP3 inflammasome, resulting in the reduction of inflammatory cytokines and amelioration of experimental diabetic nephropathy in mice [123], and also decreases the hepatic level of IL-6 (increased due to HFD intake), protecting against liver inflammation.

Metformin, a well-known drug which is in use for more than 60 years, is a biguanide derivative and is used by type-2 diabetes patients as the first line of oral therapy. It decreases the glucose absorption in the intestine, it also decreases hepatic gluconeogenesis with less risk of causing hypoglycemia, and in peripheral tissues, it increases insulin sensitivity. Moreover, it helps in reducing LDL cholesterol and encourages weight loss in obese patients. Metformin has the unique ability to activate AMP and AMPK pathways by encouraging phosphorylation at Thr-172. According to a recent cohort study, metformin was also found to minimize CVD incidents in older U.S. veterans with T2D. These results point to metformin's potential function in lowering CVD risk, and evidence indicates that combining metformin with statin therapy has an even better impact on CVD comorbidity in T2D patients [124]. A randomized controlled trial recently demonstrated a significant decrease in left ventricular hypertrophy (LVH), one of the most potent predictors for coronary artery disease, in patients with diabetes-free coronary artery disease. This study found that the ventricular mass indexed to height, left ventricular mass, body weight, and oxidative stress were significantly reduced by metformin [125].

Melatonin was first proposed as a cardioprotective substance in experimental cardiotoxicity caused by doxorubicin-induced mitochondrial degeneration. Melatonin protects against age-related vascular remodeling and heart damage from mitochondrial degeneration. Mitochondrial dysfunction caused by cardiac ischemia-reperfusion is protected by melatonin, melatonin works by inhibiting pore opening of mitochondria and *cytochrome c* release by complex I and III alterations and cardiolipin [126], and mitochondrial permeability transition pore opening. Moreover, melatonin also protects against impairment in apoptotic proteins and cardiac mitochondrial energy-metabolizing enzymes [127].

A mitochondrial targeting peptide is a novel approach that includes small cell-permeable peptide antioxidants. SS peptide, often known as Szeto-Schiller peptide, is a type of amphipathic tetra-peptide and is believed to be a breakthrough molecule that can be used to treat a wide range of mitochondrial disorders acts on the mitochondrial membrane because they have a high content of anionic phospholipid cardiolipin [128]. There are four structural composition of SS peptides namely: SS-01, SS-02, SS-31 and SS-20 [129]. The alternating aromatic residues and basic amino acids are the structural theme of these SS peptides (aromatic-cationic peptides). These SS peptides can prevent lipid peroxidation by scavenging hydrogen peroxide and peroxynitrite [130]. Recently we studies on protective the role of SS31 in type 2 diabetic mice [130,131]. SS peptides mainly work on the inner mitochondrial membrane and reduce oxidative stress. SS-02 and SS-31 possess their antioxidant properties due to their dimethyltyrosine (Dmt) residues. Moreover, SS-02 and SS-31 are very good at oxygen scavenging and inhibiting linolic acid oxidation *in vitro* [132]. Whereas tetrapeptide SS-20, where Dmt is replaced by phenylalanine group, does not possess ROS scavenging property. Numerous studies show these peptides are targeted to the inner mitochondrial membrane, due to which they can enter the matrix without disturbing the potential gradient. Recently, the data obtained by performing an in vivo study in the ALS mouse model in which SS-31 given via injection shows promising survival and motor performance [133]. Another study performed in brain tissue, giving SS-31 showed an immediate decrease in ROS levels, release of cytochrome *c* and malondialdehyde levels, and block superoxide dismutase activity. Another study performed by Yang and collaborators revealed that SS-31 and SS-20 can prevent MPTP neurotoxicity in mice [134]. Loss of dopamine and its metabolites in the striatum, as well as loss of tyrosine hydroxylase immunoreactive neurons in the substantia nigra, were entirely protected by SS-31 in a dose-dependent manner [135].

## Conclusion and future prospective

It is well established that mitochondria are more than energy-producing organelles. Many diseases such as neurodegenerative, metabolic, and a range of cancers involve altering mitochondrial function and bioenergetics in their pathology. Several studies have corroborated the link between mitochondrial dysfunction and disease pathology. The modifications in bioenergetics could be because of defects in ETC complexes, modifications in mitochondrial genes, ROS production, oxidative stress, and/or altered mitochondrial dynamics. Mitochondria are believed to be an attractive target for possible therapeutics. Various drugs, antioxidant molecules, bioactive compounds can specifically target the mitochondria and help reduce ROS or oxidative stress by attacking specific proteins and processes. The list of such combinations is not exhaustive, and further research might generate more suitable candidates. Recent Nanotechnology approaches might be used for effective drug delivery into mitochondria and possible therapeutic strategies to treat various neurodegenerative and metabolic diseases.

## Conflicts of interest

The authors have no financial or ethical conflicts of interest to report.
